# Limited transmission of carbapenem-resistant *Klebsiella pneumoniae* between animals and humans: a study in Qingdao

**DOI:** 10.1080/22221751.2024.2387446

**Published:** 2024-07-31

**Authors:** Rina Bai, Xiao Wang, Zhiyu Zou, Wenjing Zhou, Chang Tan, Yue Cao, Bo Fu, Weishuai Zhai, Fupin Hu, Yang Wang, Congming Wu, Yuanqi Zhu, Chengtao Sun

**Affiliations:** a National Key Laboratory of Veterinary Public Health and Safety, College of Veterinary Medicine, China Agricultural University, Beijing, People’s Republic of China; b Department of Clinical Laboratory, The Affiliated Hospital of Qingdao University, Qingdao, People’s Republic of China; c Institute of Antibiotics, Huashan Hospital, Fudan University, Shanghai, People’s Republic of China

**Keywords:** Carbapenem-resistant *Klebsiella pneumoniae*, human clinical, meat products, farm animals, transmission

## Abstract

Despite no carbapenem use in food animals, carbapenem-resistant *Klebsiella pneumoniae* (CRKP) perseveres within food animals, rising significant concerns regarding public health risks originating from these non-clinical reservoirs. To investigate the potential link between CRKP in food animals and its infections in humans, we conducted a cross-sectional study encompassing human clinical, meat products, and farm animals, in Qingdao city, Shandong province, China. We observed a relatively higher presence of CRKP among hospital inpatients (7.3%) compared to that in the meat products (2.7%) and farm animals (pig, 4.6%; chicken, 0.63%). Multilocus sequence typing and core-genome phylogenetic analyses confirm there is no evidence of farm animals and meat products in the clinical acquisition of *K. pneumoniae* isolates and carbapenem-resistant genes. However, potential transmission of *K. pneumoniae* of ST659 and IncX3 plasmid harbouring *bla*_NDM-5_ gene from pigs to pork and farm workers was observed. Our findings suggest a limited role of farm animals and meat products in the human clinical acquisition of *K. pneumoniae*, and the transmission of *K. pneumoniae* is more common within settings, than between them.

*Klebsiella pneumoniae* stands as a primary pathogen responsible for infections in hospitalized individuals globally, frequently causing pneumonia, wound, soft tissue, and urinary tract infections [1]. However, the rise of multidrug-resistant strains, particularly the carbapenem-resistant *Klebsiella pneumoniae* (CRKP), within clinical settings, amplifies its threat to human health [2]. As a bacterial pathogen thriving across multiple hosts, *K. pneumoniae* inhabits not only humans, but also food animals (mainly chicken and pig) and animal-producing food products (mainly chicken meat and pork) [3]. Despite the absence of carbapenem use in food animals, CRKP perseveres within food animals [4], rising significant public health concerns regarding the risk of transmission between humans and these non-clinical reservoirs. To investigate the potential link between CRKP in food animals and its infections in humans, we conducted a cross-sectional study involving human clinical samples, meat products, and farm animals in Qingdao city, Shandong province – a hub renowned for its substantial livestock production in China [5].

From October 2021 to February 2022, we collected a total of 1139 samples from hospital inpatients (*n* = 300), meat products (*n* = 112), and farm animal related (*n* = 727) (Table S1). The inpatients were recruited from the clinical laboratories of four large hospitals in Qingdao city (Figure 1(a)). Adults who had microbiological examinations were consented and included into the study. Subjects who had received antibiotic treatments in the preceding 30 days were excluded. The samples were collected from diverse sources including sputum, urine, bile, blood, ascites, bronchoalveolar lavage fluid, pus, and wound secretion (Table S1). Moreover, we selected chicken and pig farms, slaughterhouses, and meat markets that are part of a vertically integrated production system. Farm-animal-related samples were collected from five farms and two slaughterhouses, specifically focusing on pigs and chickens. The collected samples encompassed a variety of sources including animal faecal, farm worker faecal, carcasses, and environmental samples (Table S1). Additionally, meat samples were collected from the retail pork and chicken of 59 markets that sell products from the sampled slaughterhouses (Figure 1(a)).

**Figure 1.**
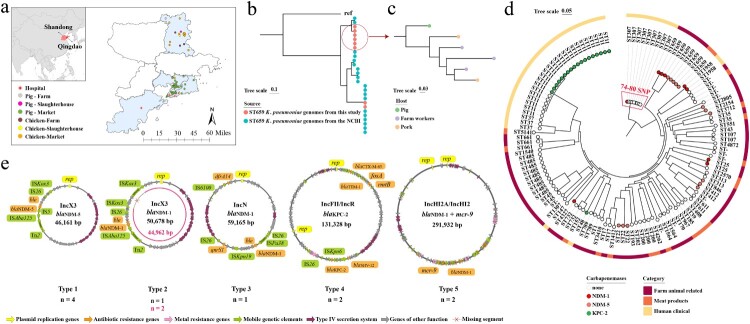
Distribution and molecular epidemiology of CRKP in human clinical, meat products, and farm animals. a. Geographical distribution of sampling site in Qingdao city. b. Phylogenetic tree of the 28 ST659 CRKP from NCBI Pathogen Detection database and our study. c. Phylogenetic tree of the six ST659 isolates with a lower mean pairwise nucleotide divergence isolated in this study. d. Phylogenetic tree of carbapenem-resistant *Klebsiella pneumoniae* and carbapenem-susceptible *Klebsiella pneumoniae* based on core-genome SNPs. Each isolate is labelled on the node with a coloured dot representing its carried carbapenemase-producing genes; the sources of the isolates are indicated by coloured squares in the outer ring. e. Genetic environment of *bla*_NDM_ or *bla*_KPC_ gene in the 12 completely sequenced plasmids, detailed information is available in Supplementary Table S2. “n” represents the count of isolates carrying this type plasmid among those long-read sequenced. The pink circle represents that this plasmid has a missing segment, as compared to the Type 2 IncX3 plasmid carrying *bla*_NDM-1_.

Analysis of the collected samples using MacConkey plates supplemented with 2 μg/mL meropenem, coupled with MALDI-TOF-MS (Bruker, Bremen, Germany), led to the identification of 42 *K*. *pneumoniae* isolates. Antimicrobial susceptibility testing using broth microdilution confirmed the resistance of all 42 *K. pneumoniae* isolates to meropenem (minimum inhibitory concentrations, MICs ≥ 4 μg/mL). We observed a relatively higher presence of CRKP among hospital inpatients (7.3%) compared to that in the farm animals (pig, 4.6%; chicken, 0.6%) (Chi-square test, *p* value < 0.05, Table S1), suggesting a difference in CRKP prevalence between humans and food-producing animals. CRKP was positively detected in all four tested hospitals, two pig farms, and two slaughterhouses, with three out of 59 meat markets also testing positive for CRKP.

To determine the population structure of CRKP isolates from inpatients (*n* = 22), meat products (*n* = 3), and farm animals (*n* = 17), we performed whole-genome sequencing on a 150 bp paired-end library for each isolate using the Illumina HiSeq X Ten System. In silico multilocus sequence typing (MLST) clustered the 42 CRKP isolates into 13 distinct sequence types (STs) (Figure S1 and Figure S2). Specifically, the 22 CRKP isolates from inpatients were clustered into four STs, while the 17 animal-related isolates grouped into eight STs, and the three isolates from animal meat into two STs (see Figure S2 and Table S2). Notably, we observe no overlap in STs between isolates from the inpatients and those from the meat products or farm animals, suggesting that hospital-acquired CRKP are not directly linked to these animal and food sources (Figure S2). However, eight isolates from the farm animals, farm workers, and meat products shared the same sequence type, ST659 (Figure 1(b); Table S2).

Sequence mapping [6] of these eight ST659 genomes, alongside all 20 ST659 *K. pneumoniae* genomes from the NCBI Pathogen Detection database (see Supplementary Methods for detail), identiﬁed 39,452 core genome SNPs (single nucleotide polymorphisms). We observed a notably lower mean pairwise nucleotide divergence of 0.03% among the six of the eight ST659 *K. pneumoniae* isolates compared to 21.43% in the 20 ST659 *K. pneumoniae* genomes from humans or animals, which are epidemiologically irrelevant (Figure 1(b)). Specifically, core-genome phylogenetic analyses revealed that the pig isolates represent the phylogenetic ancestry of the other five ST659 isolates from two pork samples and three farm workers (Figure 1(c)), suggesting potential transmission events of ST659 *K. pneumoniae* from pigs to either pork or farm workers. These findings, coupled with documented ST659 *K. pneumoniae* infections in humans [7] and our evidence from conjugation assays that ST659 *K. pneumoniae* can transfer the *bla*_NDM_-positive plasmid into clinical *K. pneumoniae* and *Escherichia coli* (Table S3), underscore the potential risk of human infections posed by ST659 *K. pneumoniae* capable of harbouring and transferring the *bla*_NDM_-positive plasmids. Moreover, core-genome phylogenetic analyses of the 42 CRKP isolates, along with 82 carbapenem-susceptible *K. pneumoniae* (CSKP) isolates identified from the same batch of samples (see Supplementary Methods for detail), reveal that isolates harbouring the *bla*_NDM_ gene within the same settings are closely related to those without *bla*_NDM_ (Figure 1(d)). This observation suggests a potential for CSKP isolates to acquire carbapenem resistance. Moreover, our conjugation assays have demonstrated that *bla*_NDM_-positive plasmid can be transferred from CRKP to *bla*_NDM_-negative counterparts (Table S3).

To further analyse the transfer of carbapenem-resistant genes via mobile genetic elements, 12 *K. pneumoniae* isolates were selected for long-read sequencing on Oxford Nanopore Technologies (Table S2; see Supplementary Methods for selection criteria). Hybrid de novo assembly produced 12 completely sequenced plasmids possessing either *bla*_NDM_ or *bla*_KPC_ gene (Figure 1(e)). We identified the *bla*_NDM-1_ gene on distinct plasmids across both human clinical and farm-animal-related isolates. Specifically, isolates from pigs (*n* = 2) and farm worker (*n* = 1) shared the *bla*_NDM-1_ on identical IncX3 plasmids, while in human isolates, it was found on IncN and IncHI2A/IncHI2 plasmids. Notably, the latter also harbouring the colistin resistance gene, *mcr-9*. In contrast, the *bla*_NDM-5_ gene, exclusively identified from isolates of pig, pork, and farm worker, was located on identical IncX3 plasmids (Table S2), providing further evidence of bacterial transmission among these hosts. The *bla*_KPC-2_ gene, identified in two ST11 human clinical *K. pneumoniae* isolates, was located on identical IncFII/IncR plasmids. Although these plasmids tested positive for the Type IV secretion system (T4SS), they showed no ability to conjugate with meropenem-susceptible porcine *K. pneumoniae* isolates (Table S3).

Despite existing evidences [8,9] and the theoretical risk of interconnected AMR reservoirs between food animals and humans, debates persist regarding the extent of AMR transmission from animals to humans [10–12]. Challenging the widely accepted One Health perspective that bacterial pathogens and their resistance genes can transmit between humans and animal sectors, the current study point to a limited role of farm animals and meat products in the clinical acquisition of *K. pneumoniae*, which is consistent with previous studies [10–12]. However, our analysis revealed that the transmission of *K. pneumoniae* among farm animals, farm workers, and food products, suggesting that the transmission of *K. pneumoniae* is much more common within settings, than between them. This is in line with previous studies as observed in *K. pneumoniae* [13], *Escherichia coli* [10]*,* and *Enterococcus faecium* [14]. As for the carbapenem-resistant genes, the distinct *bla*_NDM-1_-positive plasmids identified in human clinical and farm animal-related isolates suggest their independent evolutionary trajectories within each of their bacterial hosts. Our observation of the discrepancy in carbapenem-resistant genes between hospital isolates (primarily *bla*_KPC-2_, 19/22) and animal-related isolates (*bla*_NDM_, 20/20) further supports this point (Figure S1 and Table S2). This is consistent with previous large-scale genomic study of clinical *K*. *pneumoniae*, which found that the epidemic of CRKP in Europe is driven by nosocomial spread [15]. Despite these findings, we acknowledge the limitations in the current study. Although we collected a large number of samples from farm animals, meat products, and human clinical cases, only a small number of CRKP isolates were identified, suggesting more detailed investigations are essential for a better understanding of CRKP transmission across different settings. Moreover, the current study highlights the limited transmission of carbapenem-resistant genes within *K. pneumoniae* between animals and humans, but does not address their transmission within and among other bacterial species.

## Supplementary Material

Supplementary Materials.doc
