# Supplementary material for: Limited transmission of carbapenem-resistant Klebsiella pneumoniae between animals and humans: a study in Qingdao
Source: Emerg Microbes Infect. 2024 Jul 31;13(1):2387446. doi: 10.1080/22221751.2024.2387446 (PMC11312996; doi:10.1080/22221751.2024.2387446)
Supplement: Supplementary Materials.doc [file TEMI_A_2387446_SM5357.doc]

**Supplementary Materials**

**Supplementary** **Methods**

From October 2021 to February 2022, we collected a total of 1139 samples from hospital inpatients (n = 300), meat products (n = 112), and farm animal related (n = 727) (Table S1). Our sampling strategy covered an entire meat-producing chain (from live animal breeding, to slaughtering, and retail) to human. We selected farms, slaughterhouses, and markets that are part of a vertically integrated production system: live pigs and chickens from the sampled farms are programmed slaughtered in the sampled slaughterhouses, and the resulting raw meat would transport to the sampled markets for sale. The human clinical samples were collected from four large comprehensive hospitals located in near the sampled markets.

For all samples, a pre-enrichment step in the LB broth was performed in a constant temperature shaker at 37℃ overnight. The enrichments were then inoculated onto MacConkey plates supplemented with 2 μg/mL meropenem (a concentration below the meropenem resistance breakpoint was used to ensure that no meropenem-resistant isolates were missed). Suspected colonies were picked based on colony morphotypes and repeatedly streaked on the MacConkey agar to obtain pure isolates. Bacterial species' identification was confirmed by MALDI-TOF mass spectrometry (BrukerDaltonik GmbH, Bremen, Germany). Antimicrobial susceptibility testing using broth microdilution in Mueller-Hinton broth confirmed the resistance of all 42 *K. pneumoniae* isolates to meropenem (minimum inhibitory concentrations, MICs ≥ 4 μg/mL, CLSI, M100-S31). Additionally, all samples from which carbapenem-resistant *K. pneumoniae* was not isolated were screened for carbapenem-susceptible *K. pneumoniae* using non-selective MacConkey plates in combination with MALDI-TOF mass spectrometry. Subsequently, antimicrobial susceptibility testing was performed to confirm their minimum inhibitory concentrations (MICs), resulting in a total of 82 strains of carbapenem-susceptible *K. pneumoniae*.

The Genomic DNA of isolates were extracted using a HiPure Bacterial DNA Kit (Magen, Guangzhou, China) following the protocols described by the manufacturer. Samples were sequenced on the Illumina HiSeq X Ten System with a read length of 150 bp, paired-end. Genome assembly [1] was conducted with SPAdes, version 3.9.0. Oxford nanopore MinION sequencing was conducted to obtain the complete genome sequences of 12 carbapenem-resistant *K. pneumoniae* isolates, using the SQK-RBK004 sequencing kit and ﬂowcell R9.5. Hybrid assembly of Illumina and nanopore sequencing reads was constructed using Unicycler v 0.3.0 [2].

The 12 *K. pneumoniae* isolates were deliberately chosen for the following reasons: 1) three *bla*_NDM-1_-positive *K. pneumoniae* isolates from human clinical samples were selected to determine the potential mobile genetic elements harboring *bla*_NDM-1_ gene. 2) four *bla*_NDM_ - positive ST659 *K. pneumoniae* isolates were selected as genome analysis of the ST659 isolates suggested potential transmission events of ST659 *K. pneumoniae* from pigs to either pork or farm workers. 3) an additional five isolates (two from humans, one from pork, and two from live animals) were selected to gain a relatively comprehensive understanding of the plasmids carrying carbapenem-resistant genes.

We downloaded all *Klebsiella pneumoniae* assemblies from the NCBI Pathogen Detection database (https://www.ncbi.nlm.nih.gov/pathogens/isolates/) as of December 20, 2023 (n=63,135). Multilocus sequence typing (MLST) using Kleborate v2.3.2 [3] identified a total of 20 ST659 *K. pneumoniae* genomes. These were included for comparison with the eight ST659 *K. pneumoniae* isolates identified in the current study.

The assembled data were screened against the ResFinder database using ABRicate v1.0.1 to detect acquired AMR genes. Assembled draft genomes were annotated using the rapid prokaryotic genome annotation tool Prokka v1.5 [4]. The MLST of the isolates was determined using the Kleborate v2.3.2, and minimum spanning trees of all sequence types was constructed in the BioNumerics v7.6 according to correlations among alleles. The phylogenetic tree based on the multiple core-genome SNP alignments was constructed using Parsnp in the Harvest package (version 1.1.2) [5] and visualized using the Interactive Tree of Life (iTOL). The geographical distribution of all sampling sites was visualized using QGIS 3.16 based on GPS information.


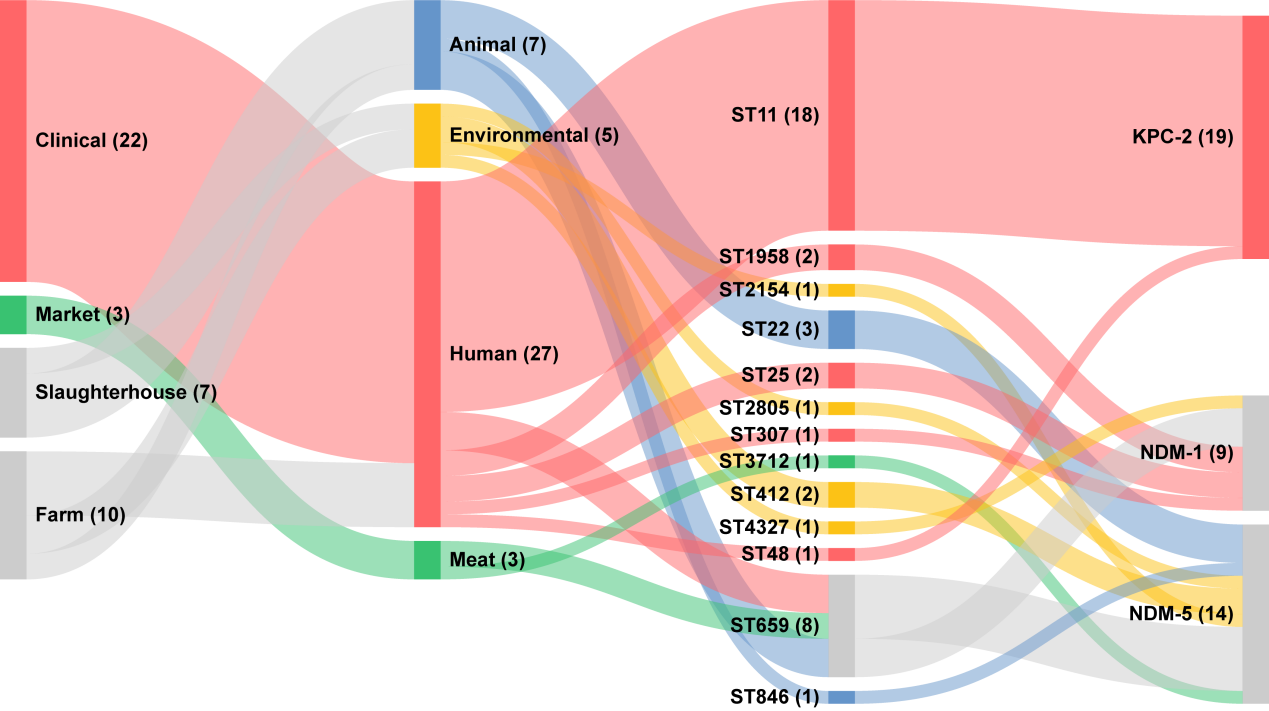


**Supplementary Figure S1. Sankey Diagram Illustrating the Relationships Among Categories, Hosts, Sequence Types (STs), and Carbapenemase-Producing Genes of the 42 Carbapenem-Resistant *Klebsiella pneumoniae* Isolates.** The diameter of the line is proportional to the number of isolates and is labelled at the consolidation points. Lines are colored based on the host types; mixture from various host types are indicated in gray.


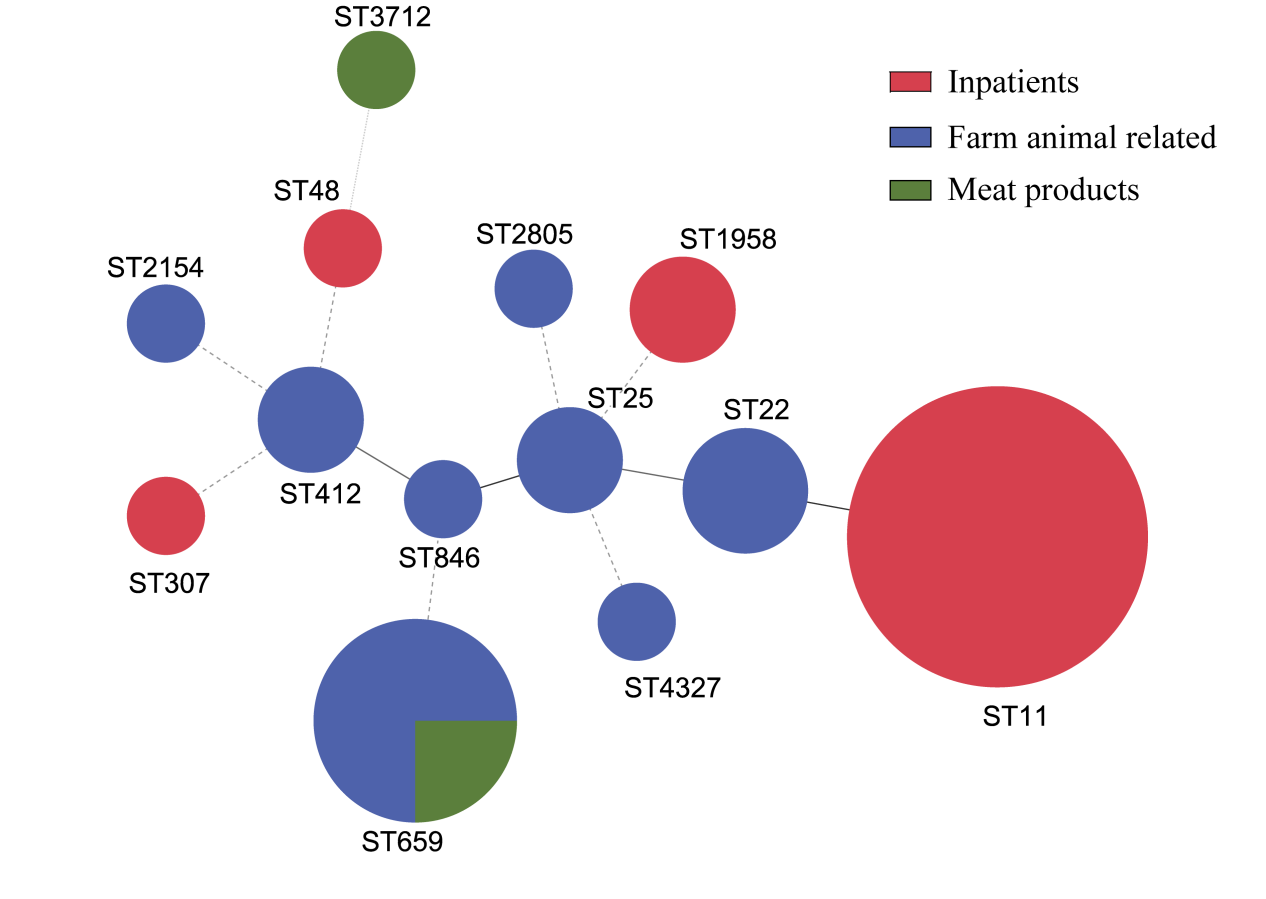


**Supplementary Figure S2. Minimum Spanning Tree of 42 Carbapenem-Resistant Klebsiella pneumoniae Isolates.** Each circle represents a different sequence type. The size of each circle is proportional to the number of isolates, and the circles are colored based on their collection sources.

**Supplementary Table S3. Conjugation of Carbapenemase-Producing Plasmids from CRKP to Carbapenem-Susceptible Isolates Across Different Hosts and STs.**

| **Donor strains** | | | | | **Recipient strains** | | | | | **Conjugation frequency**  **(mean ± SD)** |
| --- | --- | --- | --- | --- | --- | --- | --- | --- | --- | --- |
| **ID** | **ST** | **Host** | **Resistant phenotype** | **Tested plasmids** | **ID** | **Species** | **ST** | **Host** | **Resistant phenotype** |
| 21QDDZ10 | ST659 | Pig | Meropenem | IncX3 (*bla*NDM-1) | 21QDRDZ6 | *E. coli* | ST2540 | Human | Tigecycline | 2.67E-06 ± 1.58E-06 |
| 21QDDZ10 | ST659 | Pig | Meropenem | IncX3 (*bla*NDM-1) | 21QDRDZ41 | *K. pneumoniae* | ST629 | Human | Tigecycline | 8.82E-07 ± 4.55E-07 |
| 21QD5MM2 | ST659 | Pork | Meropenem | IncX3 (*bla*NDM-5) | 21QDRDZ6 | *E. coli* | ST2540 | Human | Tigecycline | 3.95E-06 ± 7.64E-08 |
| 21QD5MM2 | ST659 | Pork | Meropenem | IncX3 (*bla*NDM-5) | 21QDRDZ41 | *K. pneumoniae* | ST629 | Human | Tigecycline | 1.53E-06 ± 8.61E-07 |
| 21QD2ABFB1 | ST659 | Worker | Meropenem | IncX3 (*bla*NDM-5) | 21QDRDZ6 | *E. coli* | ST2540 | Human | Tigecycline | 5.57E-06 ± 1.79E-06 |
| 21QD2ABFB1 | ST659 | Worker | Meropenem | IncX3 (*bla*NDM-5) | 21QDRDZ41 | *K. pneumoniae* | ST629 | Human | Tigecycline | 8.58E-06 ± 5.78E-06 |
| 21QD2ABZ15 | ST864 | Pig | Meropenem | IncX3 (*bla*NDM-5) | 21QDRDZ6 | *E. coli* | ST2540 | Human | Tigecycline | 1.11E-06 ± 5.20E-07 |
| 21QD2ABZ15 | ST864 | Pig | Meropenem | IncX3 (*bla*NDM-5) | 21QDRDZ41 | *K. pneumoniae* | ST629 | Human | Tigecycline | 5.44E-06 ± 6.01E-06 |
| K38 | ST307 | Human | Meropenem | IncN (*bla*NDM-1) | 4427 | *K. pneumoniae* | ST412 | Pig | Colistin | 2.51E-06 ± 7.31E-07 |
| K10 | ST11 | Human | Meropenem | IncFII/IncR (*bla*KPC-2) | 471 | *K. pneumoniae* | ST39 | Pig | Tigecycline | Conjugation failed |
| K110 | ST1958 | Human | Meropenem | IncHI2A/IncHI2 (*bla*NDM-1+*mcr-9*) | 4427 | *K. pneumoniae* | ST412 | Pig | Colistin | 1.27E-06 ± 1.61E-07 |
| K110 | ST1958 | Human | Meropenem | IncHI2A/IncHI2 (*bla*NDM-1+*mcr-9*) | J53 | *E. coli* | / | / | Sodium azide | 2.63E-06 ± 6.08E-07 |

**Reference**

[1] Bankevich A, Nurk S, Antipov D, et al. SPAdes: a new genome assembly algorithm and its applications to single-cell sequencing. J Comput Biol. 2012 May;19(5):455-77.

[2] Wick RR, Judd LM, Gorrie CL, et al. Unicycler: Resolving bacterial genome assemblies from short and long sequencing reads. PLoS Comput Biol. 2017 Jun;13(6):e1005595.

[3] Lam MMC, Wick RR, Watts SC, et al. A genomic surveillance framework and genotyping tool for Klebsiella pneumoniae and its related species complex. Nat Commun. 2021 Jul;12(1):4188.

[4] Seemann T. Prokka: rapid prokaryotic genome annotation. Bioinformatics. 2014 Jul;30(14):2068-2069.

[5] Treangen TJ, Ondov BD, Koren S, et al. The Harvest suite for rapid core-genome alignment and visualization of thousands of intraspecific microbial genomes. Genome Biol. 2014 Nov;15(11):524.
